# Improving Services for HIV-Exposed Infants in Zambia and Cameroon Using a Quality Improvement Collaborative Approach

**DOI:** 10.9745/GHSP-D-20-00550

**Published:** 2021-06-30

**Authors:** Gillian Dougherty, Tihnje Abena, Jean Pierre Abesselo, Jeane Ngala Banda, Tjek Paul Biyaga, Rodrigo Boccanera, Mary Adetinuke Boyd, Mesmey Ebogo, Leoda Hamomba, Suzanne Jed, Zeh Florence Kakanou, Prisca Kasonde, Siphiwe Chilungu Kasonka, Rachael Lungwebungu, Caitlin Madevu-Matson, Magdalene Mange Mayer, Mukuka Mwamba, Milembe Panya, Paul Sakanda, Fatima Tsiouris, Lauren Walker, Miriam Rabkin

**Affiliations:** a ICAP at Columbia University, Columbia University Mailman School of Public Health, New York, NY, USA.; b ICAP at Columbia University, Yaoundé, Cameroon.; c Ministry of Health, Lusaka, Zambia.; d Ministry of Health, Yaoundé, Cameroon.; e Health Resources and Services Administration, Rockville, MD, USA.; f Centers for Disease Control and Prevention, Lusaka, Zambia.; g ICAP at Columbia University, Lusaka, Zambia.; h Centers for Disease Control and Prevention, Yaoundé, Cameroon.; i ICAP at Columbia University, Dar es Salaam, Tanzania.

## Abstract

**Introduction:**

Early infant diagnosis (EID) and rapid antiretroviral therapy (ART) initiation are lifesaving interventions for HIV-infected infants. In Cameroon and Zambia, EID coverage for HIV-exposed infants (HEIs) is suboptimal and the time to ART initiation for infants infected with HIV often exceeds national standards despite numerous policy and training initiatives.

**Methods:**

ICAP at Columbia University supported the Cameroon and Zambia Ministries of Health (MOHs) and local partners to implement quality improvement collaboratives (QICs) to improve EID coverage and ART initiation at 17 health facilities (HFs) in Cameroon (March 2016 to June 2017) and 15 HFs in Zambia (March 2017 to June 2018). In each country, MOH led project design and site selection. MOH and ICAP provided quality improvement training and monthly supportive supervision, which enabled HF teams to conduct root cause analyses, design and implement contextually appropriate interventions, conduct rapid tests of change, analyze monthly progress, and convene at quarterly learning sessions to compare performance and share best practices.

**Results:**

In Cameroon, EID testing coverage improved from 57% (113/197 HEIs tested) during the 5-month baseline period to 80% (165/207) in the 5-month endline period. In Zambia, EID testing coverage improved from 77% (4,773/6,197) during the 12-month baseline period to 89% (2,144/2,420) during the 3-month endline period. In a comparison of the same baseline and endline periods, the return of positive test results to caregivers improved from 18% (36/196 caregivers notified) to 86% (182/211) in Cameroon and from 44% (94/214) to 79% (44/56) in Zambia. ART initiation improved from 44% (94/214 HIV-infected infants) to 80% (45/56) in Zambia; the numbers of HIV-infected infants in Cameroon were too small to detect meaningful differences.

**Conclusions:**

QICs improved coverage of timely EID and ART initiation in both countries. In addition to building quality improvement capacity and improving outcomes, the QICs resulted in a “change package” of successful initiatives that were disseminated within each country.

## Abstract (precis)

To bridge the gap between what is known and what is done, quality improvement collaboratives (QICs) enable health programs to rapidly address quality challenges at scale. Two QICs in Cameroon and Zambia improved coverage of early infant HIV testing and initiating antiretroviral therapy in HIV-exposed infants. The QIC approach empowers health care workers to design solutions tailored for their specific settings.

## Teaser

**Key Findings:**

- Although the designs of the QICs varied to fit local contexts in Cameroon and Zambia, they used similar indicators and methods, and each resulted in improvement of testing coverage and timely return of test results for HIV-exposed infants.
- The social dynamics of the QIC approach enhanced the sense of shared purpose and community, fostered teamwork and friendly competition, and built leadership support while creating an internal enabling environment at the facility level. The quarterly learning sessions, monthly data collection, and ongoing mentoring for quality improvement led to significant improvements that were sustained for the life of the intervention.

**Key Implications:**

- The use of QIC methodology empowered health care workers to design solutions tailored to their specific settings, and each QIC resulted in a “change package” of successful initiatives that were disseminated within each country.
- Health sector policy makers should consider institutionalizing the QIC approach and fostering its targeted implementation to address refractory quality challenges, including the inclusion of QIC methods in national policies, guidelines, training, and monitoring systems.

Résumé en français à la fin de l'article.

## INTRODUCTION

Since the release of the Joint United Nations Programme on HIV/AIDS (UNAIDS) Fast Track declaration in 2015, the global community has worked to achieve HIV epidemic control by 2030 by ensuring that at least 95% of people living with HIV are aware of their status, 95% of those aware of their status are linked to antiretroviral therapy (ART), and 95% of those on ART have achieved viral suppression.^1^^,^^2^ Although many countries have made remarkable progress toward reaching these goals for adults, the same cannot be said for infants and children. In 2018, for example, the global community achieved only 59% of pediatric ART coverage targets.^3^

High-quality national programs are essential to prevent early mortality due to pediatric HIV, which peaks at 3–4 months^4^ and approaches 50% by 2 years of age.^5^ Unfortunately, the health systems required to deliver HIV testing and treatment services to infants and children are lacking in many settings, and for two-thirds of HIV-infected children in Africa, Asia, and the Americas, HIV is only diagnosed when the children have advanced immunodeficiency, leading to high rates of preventable morbidity and mortality.^6^

**FIGURE 1:**
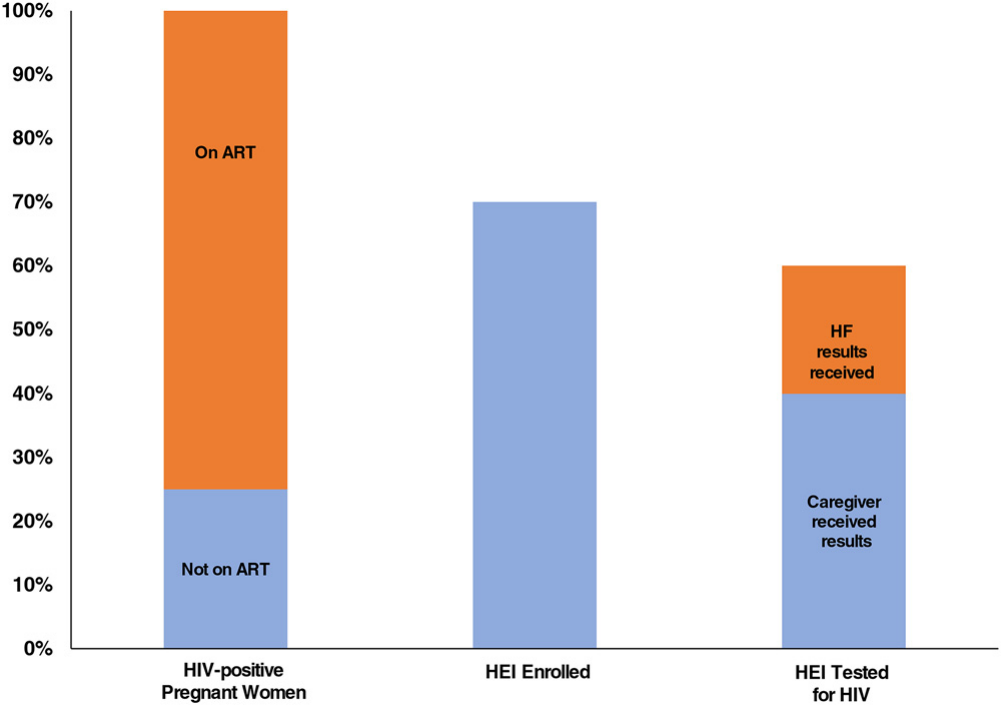
Illustrative Cascade of Interventions Needed to Prevent Mother-to-Child Transmission of HIV, Diagnose Infants With HIV, and Link Infants to Antiretroviral Therapy Abbreviations: ART, antiretroviral therapy; EID, early infant diagnosis of HIV; HEI, HIV-exposed infants; HF, health facility.

The interventions needed to prevent mother-to-child transmission (PMTCT) of HIV and swiftly identify HIV-infected infants and link them to ART can be conceptualized as a cascade of services (Figure 1). Health systems must consistently and correctly identify and engage HIV-infected pregnant women, provide ART for those not already on treatment, and deliver a package of services to their HIV-exposed infants (HEIs). These early infant diagnosis (EID) interventions include maternal counseling, HIV testing before 8 weeks of age, rapid return of results to parents/caretakers and treating clinicians, and prompt ART initiation for HIV-infected infants.

Cameroon and Zambia are both facing generalized HIV epidemics, with adult HIV prevalence of 3.1% and 11.5%, respectively.^7^^,^^8^ Although Zambia's PMTCT and EID coverage are substantially higher than those in Cameroon (Table 1), neither country has optimized their PMTCT-EID cascade, and substantial gaps remain in HIV services for infants born to HIV-infected women.^9^

**TABLE 1.** HIV Prevalence, Antiretroviral Therapy Coverage, and Early Infant Diagnosis of HIV Coverage in Cameroon and Zambia, 2018 Data

	Cameroon, %	Zambia, %
Adult HIV prevalence (15–49 years)	3.6	11.3
HIV incidence (all ages)	1.02	2.97
Adults on ART (ages 15–49 years)	55	78
Children on ART (ages 0–14 years)	24	79
Pregnant women accessing ART for PMTCT	80	95
EID coverage (infants tested for HIV at <8 weeks of age)	61	71

Abbreviations: ART, antiretroviral therapy; EID, early infant diagnosis; PMTCT, prevention of mother-to-child transmission of HIV.

Both Cameroon and Zambia have national PMTCT and EID policies, strategies, guidelines, training curricula, and systems for supportive supervision, supply chain management, and program monitoring and evaluation. Despite these national efforts, consistent implementation of EID services is lacking.^10^^,^^11^ While variation exists within the quality and/or effectiveness of the implementation of these health system inputs, bridging this ongoing “know-do gap” has become critically important for improving patient outcomes.

The know-do gap between established standards of care (what we know) and the ability of health systems to produce improved outcomes (what we do) has become an area of focus for country HIV programs and international donors.^12^ The use of quality improvement (QI) methodologies has been successful in closing challenging know-do gaps such as those seen in Cameroon and Zambia.^13^^–^^15^ In particular, the QI collaborative (QIC) approach has shown great promise in improving health programs in low-resource settings.^16^^–^^21^

The QIC approach has shown great promise in improving health programs in low-resource settings.

To bridge the EID know-do gap in Cameroon and Zambia, ICAP partnered with the Ministry of Health (MOH) in each country, donors, and implementing partners to design and implement the QIC projects to improve 3 key steps in the cascade: EID coverage, timely return of HIV test results, and rapid ART initiation for infants found to be HIV infected.

## METHODS

### QIC Design

#### QIC Methodology

**FIGURE 2:**
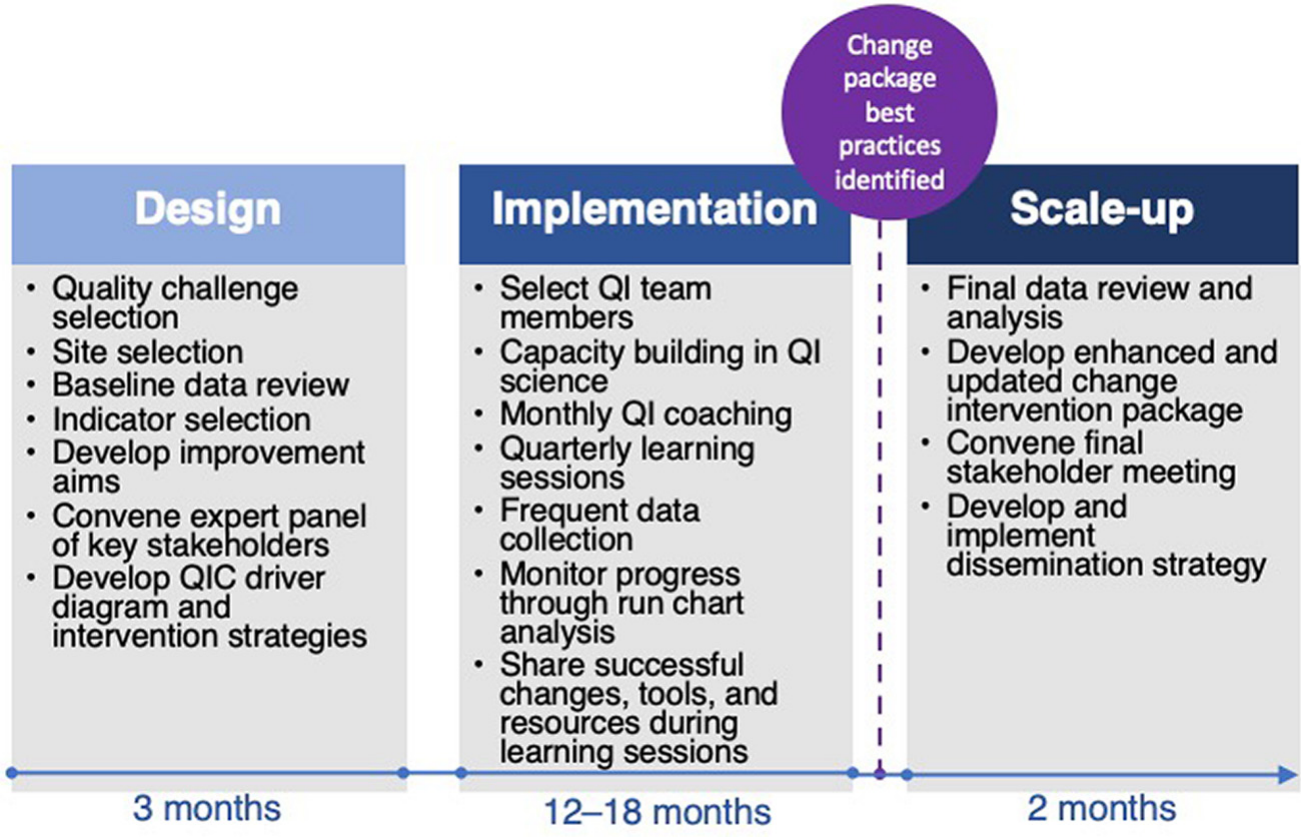
Quality Improvement Collaborative Approach Used to Improve Early Infant Diagnosis and Antiretroviral Therapy Initiation and Health Facilities in Cameroon and Zambia^a^ Abbreviations: QI, quality improvement; QIC, quality improvement collaborative.^a^ Adapted from the Institute for HealthCare Improvement Breakthrough Series.

ICAP and its MOH partners used a well-established QIC approach based on the Institute for Healthcare Improvement (IHI) known as the Breakthrough Series, which supports multiple health facilities (HFs) to address the same quality challenge at the same time to achieve rapid, measurable, and sustained improvements.^18^^,^^21^ The QIC approach begins with convening key MOH stakeholders to identify the specific health care quality challenge and kick-start the design of the QIC (Figure 2). The partners collaborate to select project HF sites and develop shared QIC aims (targets), indicators, and a measurement strategy. Baseline assessments inform the development of appropriate and specific QIC aim statements and provide data with which to monitor and assess QIC progress.^21^^,^^22^

Multidisciplinary QI teams are established at each participating HF with various cadres of HF staff; after baseline training and orientation, each QI team is supported to identify contextually appropriate interventions and perform rapid iterative tests of change using the Model for Improvement and its plan-do-study-act (PDSA) cycles.^13^ The PDSA approach helps teams test changes and see whether they yield improvements; the results are then analyzed to decide whether to implement, modify, or abandon the proposed intervention. If the intervention does not achieve the desired results, it is modified or replaced and the PDSA cycle is repeated.^23^

The PDSA approach helps teams assess changes and possible improvements; the results guide whether to implement, modify, or abandon the proposed intervention.

HFs then come together for quarterly meetings, in which they compare progress and share interventions and innovations.^22^ In addition to building QI capacity and improving targeted outcomes, QICs often develop a “change package” of tools, strategies, and best practices that can be shared across teams, scaled up, and widely disseminated. This approach has been shown to produce large-scale improvements in both high- and low-resource settings.^19^^,^^24^^,^^25^

In both Cameroon and Zambia, ICAP supported the national MOHs to design the QICs in collaboration with a panel of expert stakeholders including MOH leaders, the Health Resources and Services Administration (HRSA), the Centers for Disease Control and Prevention (CDC), and local implementing partners. MOHs had final approval of site selection, indicators, and other key project elements. Because of this collaborative MOH-led design process, the 2 QICs were slightly different in their design and focus (Table 2).

**TABLE 2.** Design of Quality Improvement Collaborative Indicators Used in Cameroon and Zambia to Improve Early Infant Diagnosis of HIV and Antiretroviral Therapy Initiation

	Cameroon	Zambia
EID testing coverage and timing	Percentage of eligible HEIs who received EID testing at <8 weeks of age	Percentage of HEIs tested who received EID testing at <8 weeks of age
	Number of eligible HEIs tested for HIV at >8 weeks of age	Number of HEIs tested for HIV at >8 weeks of age
	Number of eligible HEIs tested each month	Number of HEIs tested each month
Test results return to caregiver	Percentage of HEIs tested who were identified as HIV infected each month	Percentage of HEIs tested who were identified as HIV infected each month
	Percentage of all HEI HIV test results returned to caregiver this month	Percentage of HEIs identified as HIV infected whose caregivers received their results this month
Turnaround time	Mean time from health facility receiving HEI HIV test results from national laboratory and sharing with caregiver	Mean time between positive EID HIV test result and ART initiation
ART initiation	Percentage of HIV-infected infants newly initiated on ART each month	Percentage of HIV-infected infants initiated on ART each month
		Percentage of HIV-infected infants initiated on ART the same day their positive test was received each month
		Percentage of HIV-infected infants initiated on ART within 2 weeks of receiving positive test results each month

Abbreviations: ART, antiretroviral therapy; EID, early infant diagnosis; HEI, HIV-exposed infant.

#### Cameroon QIC

Between March 2015 and June 2017, ICAP, HRSA and CDC in Cameroon supported the Cameroon MOH National AIDS Control Department to design and implement a 15-month QIC to improve EID and ART initiation at 17 ICAP-supported sites in Centre and Littoral Regions. Included in the 17 participating HFs were 3 general hospitals, 9 district hospitals, 3 integrated health centers, and 2 ambulatory care centers. The primary focus of the QIC was to reduce the turnaround time of important steps along the testing cascade, with a focus on the return of EID test results to caregivers. The QIC also measured rates and speed of ART initiation among HEIs found to be HIV infected. Criteria for site selection were developed by MOH and included all HFs directly supported by ICAP Cameroon in Centre and Littoral Regions that had PMTCT and EID services and MOH staff available to participate on a QI team.

#### Zambia QIC

Between March 2017 and June 2018, in collaboration with HRSA, CDC in Zambia, MOH, the Lusaka Province Health Office, and the Centre for Infectious Disease Research in Zambia, ICAP implemented a QIC focused on improving EID and ART initiation (on the same day as the positive HIV test result) among HIV-infected infants at 15 HFs in Lusaka Urban District. The mix of HF types included 5 district hospitals, 9 health centers, and 1 ambulatory care health post. The QIC also measured EID coverage and turnaround time between critical cascade steps.

The Zambia MOH National HIV/AIDS/STI/TB Council (NAC) supports a well-established performance and QI strategy with successful QI project implementation dating back to 1991.^26^^,^^27^ QI leaders embedded in NAC facilitated the design and implementation efforts, providing critical leadership of all aspects of the QIC.

### QIC Implementation

#### Baseline Assessment

Retrospective aggregate monthly data were abstracted from HF registers by HF staff using a standardized paper-based tool. HF and ICAP staff then entered these baseline data into a standalone District Health Information System (DHIS2)^28^ database and conducted descriptive analyses. In Cameroon, the baseline assessment included 5 months of data (October 2015 to February 2016). In Zambia, the baseline assessment included 12 months of data (January to December 2016).

#### Staffing, Training, and Project Launch

Each participating HF assembled a multidisciplinary QIC team, including maternal-child health and ART nurses, medical officers, laboratory technicians, lay counselors, and/or HF managers. Each team attended an initial 1-week workshop, at which ICAP provided training on QI tools and methods and QIC indicators, as well as refresher training on national EID and infant ART guidelines. In Zambia, materials from the national QI curriculum were used throughout the 1-week workshop. In both countries, Cameroonian and Zambian national QI leaders served as expert trainers and facilitators. Using their baseline data, HF teams used process maps, fishbone diagrams, and driver diagrams to identify root causes associated with low EID coverage, loss to follow-up, and low rates of ART initiation for HIV-infected infants. Teams then selected and prioritized interventions (“change ideas”) tailored to their HF and designed to produce rapid improvement.

Each HF assembled a multidisciplinary QIC team, including nurses, medical officers, laboratory technicians, lay counselors, and/or HF managers.

Institutionalizing QI and building local QI capacity at the central and facility levels are critical to achieving and maintaining epidemic control. Evidence shows that country MOH QI capacity building requires action-based learning and ongoing skill building of QI systematic approaches to produce self-sustaining and scalable change.^29^^,^^30^ Effective QI training requires experiential learning whereby both staff and leaders within QI teams can directly apply knowledge within the context of ongoing QI projects using the currently available resources. In both countries, the initial QI workshops were designed to provide QI teams composed of MOH central, district, and facility staff with the opportunity to apply QI theory to everyday work directly at each HF through the use of common practical approaches and tools.^27^^,^^28^

#### Supportive Supervision and Quarterly Learning Sessions

Each month, in collaboration with local partners and MOH, ICAP provided on-site supportive supervision, QI coaching, and mentoring on data collection and analysis to the QIC teams at each HF. The supportive supervision visits were led through each MOH district health team per their routine HIV program supervision schedule. The visits provided MOH QI mentors with the opportunity to help HF teams identify successful change ideas and link progress to real-time data in DHIS2. The use of PDSA cycle implementation is the heart of QI and is well embedded into the QIC approach.^29^ The PDSA framework is grounded in continuous learning and guides thoughtful team-based actions. The tool is intended to help health care workers (HCWs) implement and practically assess if a desired change is leading to improvement in real time.^31^ The process typically involves conducting several different “tests of change” followed by systematically documenting, analyzing, adapting, retesting, and re-evaluating the iterative PDSA cycles using the PDSA worksheets as a tool.^32^ Although this essential QI methodology appears straightforward and clear, recent literature suggests that real-world application is more complex, with wide variation in how changes are tested and challenges that cause teams to implement changes with lack of rigor and consistency.^33^^,^^34^ General parameters and group consensus for the identification of successful change interventions included a demonstration of HCW compliance with implementation, staff satisfaction with the intervention, improvements in monthly data, and predictions about sustainability.

Although the essential QI methodology appears straightforward and clear, the real-world application is more complex.

Quarterly follow-up learning session workshops were convened for each project. QIC teams reported their progress using the shared indicators and described their PDSA cycle results. These peer-to-peer meetings provided HF staff with the opportunity to share lessons learned, best practices, failed ideas, and successful tools. These meetings also enabled each QIC team to benchmark their progress against other HFs via friendly competition and to communicate with senior leaders regarding their experiences while advocating for above-site, district level interventions. Each team was also provided with time to plan for the next quarter's activities. During the final learning session, the highest-performing teams were recognized with awards.

#### Data Collection and Analysis

Each month, HF QI teams collected aggregate anonymized data, shared the data with ICAP using standardized paper forms, and plotted their data on annotated run charts. ICAP staff entered the data into a dedicated DHIS2 database that was systematically reviewed every month for data quality. If errors were identified, HFs were contacted to obtain the correct information. Microsoft Excel 2012 was used to generate monthly descriptive statistics and graphs showing progress toward targets for each HF as well as the performance of the collaborative as a whole. QIC indicator performance was assessed for each HF during the implementation period and the range, mean, and median across HFs was calculated. In addition to descriptive statistics, project baseline performance was compared with performance during the final endline period (3–5 months) of the intervention period using the chi-squared test of independence. Run charts were constructed by the QIC HF teams, who entered the data every month. Run chart rules of analysis were utilized to monitor processes, measure performance to the aim, and measure the impact of change interventions.^35^^,^^36^

#### Dissemination of Successful Change Ideas

After 15 months of implementation, each project convened a final stakeholders' meeting in which experiences, results, and the package of successful changes and best practices (Table 3) were shared with MOH, regional- and district-level health leaders, implementing partners, and representatives of the U.S. President's Emergency Plan for AIDS Relief (PEPFAR) agencies. During this final meeting, higher-performing HFs were invited to present their project results and change interventions, and MOHs and their partners developed strategies for sustainability, scale-up, and spread of improvements to other parts of their respective country. Funding for each project was completed at the end of the implementation of each QIC. The MOHs in Zambia and Cameroon will lead the scale-up and institutionalization of the QICs in their respective countries.

**TABLE 3.** Cameroon and Zambia Change Ideas Shared With Stakeholders After 15 Months of Implementation of a Quality Improvement Collaboration to Improve Early Infant Diagnosis of HIV and Antiretroviral Therapy Initiation

Health care worker capacity building	Provide on-the-job mentorship to optimize PMTCT services
	Demonstrate proper DBS sample collection for HF staff in MCH, labor, and postnatal wards, as needed
	Orient new lay counselors on standard documentation and register completion
	Pair lay counselors based on experience levels to enable peer-to-peer learning
Data quality and documentation	Review registers monthly and provide refresher trainings to fill gaps, as needed
	Reinforce proper documentation practices among all MCH lay counselors
	Enlarge and display national register standard operating procedures
	Assign nurses to supervise lay counselor documentation practices
	Confirm and update caregiver contact information with every visit
	Conduct quarterly reviews for data quality in relevant registers
Client and family education and engagement	Provide targeted, one-on-one health education talks to HIV-infected mothers
	Deliver HF invitations to increase male partner involvement/participation in MCH services
	Recruit and engage mentor mothers to provide health education from the peer perspective
	Provide one-on-one health information and counseling to HIV-infected mothers regarding male involvement in care and the importance of disclosure
	Male partner engagement during ANC visits for all education provided
	Introduce a “care buddy” to increase retention in care where clients attend treatment preparation sessions with a friend, family member, or support person to help with treatment adherence
Workflow process improvements	Develop and use DBS tracking forms between ANC and laboratory departments
	Develop and display a flowchart to illustrate MOH standard of care
	Prioritize immediate action on positive HIV test results received from laboratory
	Develop interfacility communication system to enable confirmed patient transfers
	Assign HIV test (DBS) stock management focal persons
	Active HEI case finding through retrospective ANC chart review and follow up
	Engage the facility-based “linkage officer” to facilitate communication of DBS HIV test results with MCH staff
	Screen postnatal discharge cards upon arrival at postnatal care for HIV testing
	Develop and use a tracking list to follow up on missing DBS results
	Designate specific days to prioritize the provision of EID and ANC services
Community engagement	Engage community-based volunteer peer mothers in active tracking and follow-up
	Introduce geographic HIV-infected pregnant women social networks
	Convene sensitization meetings to engage religious leaders in the community
	Engage safe mother action groups in tracking and follow-up activities

Abbreviations: ANC, antenatal care; ART, antiretroviral therapy; DBS, dried blood sample; EID, early infant diagnosis; HEI, HIV-exposed infant; HF, health facility; MCH, maternal child health; MOH, Ministry of Health; PMTCT, prevention of mother-to-child transmission of HIV.

### Ethical Review

Both projects received a nonresearch determination from the Columbia University Institutional Review Board (Cameroon: AAAQ5055 and Zambia: AAAR2850) and the HRSA Office of Planning, Analysis, and Evaluation. The Cameroon Ministry of Public Health Division of Operational Research granted a Letter of Exemption, and the University of Zambia Biomedical Research Ethics Committee granted a waiver of ethics review.

## RESULTS

In each country, all HFs participated throughout the QIC and all learning sessions included representatives from each HF. ICAP and MOH partners made 272 supportive supervision visits over 15 months (March 2016 to June 2017) in Cameroon and 235 supportive supervision visits over 15 months (March 2017 to May 2018) in Zambia. Root cause analyses revealed that typical barriers to implementation of the EID cascade included staff knowledge deficits, unclear roles and responsibilities, process breakdowns, and systems bottlenecks. In response, the QIC teams tested interventions related to test result management, improved staff and client education, staffing modifications, workflow process modifications, commodity management, documentation, and data quality improvements (Table 3).

The PDSA method provided HCWs with a simple algorithm for implementing, testing, and adapting improvement interventions. The multidisciplinary HF teams worked together to develop and test contextually specific interventions tailored for their sites and communities. For example, a few QIC teams in Cameroon identified the common practice of pediatric caregivers providing incorrect contact information to nurses, which hindered necessary follow-up. In response, these teams developed enhanced patient education, including one-on-one counseling and focus groups, to build trust between clients and staff and to explain the importance of HIV testing at recommended intervals, as well as the rationale behind requesting contact information. As observed in other settings, QIC leadership teams found that while PDSA is simple in theory, application in real life can be somewhat complicated and requires QI teams to thoughtfully dissect their data while drawing shared conclusions about the intervention and its subsequent “success” or “failure” with progress to the QIC aim.

The PDSA method provided HCWs with a simple algorithm for implementing, testing, and adapting improvement interventions.

The time required for HF teams to become more independent with PDSA cycle management ranged from 6 to 9 months. During site support visits, MOH leadership and QIC teams initially observed common challenges with QI team PDSA cycle implementation including inadequate planning of the “who, what, where, when, and how” of the change; poor documentation; implementation on too large of a scale; failure to secure site-level leadership buy-in; poor data quality; and poor communication between team members. Over time, HF teams mastered the skills needed to independently identify, implement, and test change ideas.

Table 3 outlines the most successful change interventions identified through group consensus and QI team professional expertise using data and subjective assessment of each intervention.

**FIGURE 3:**
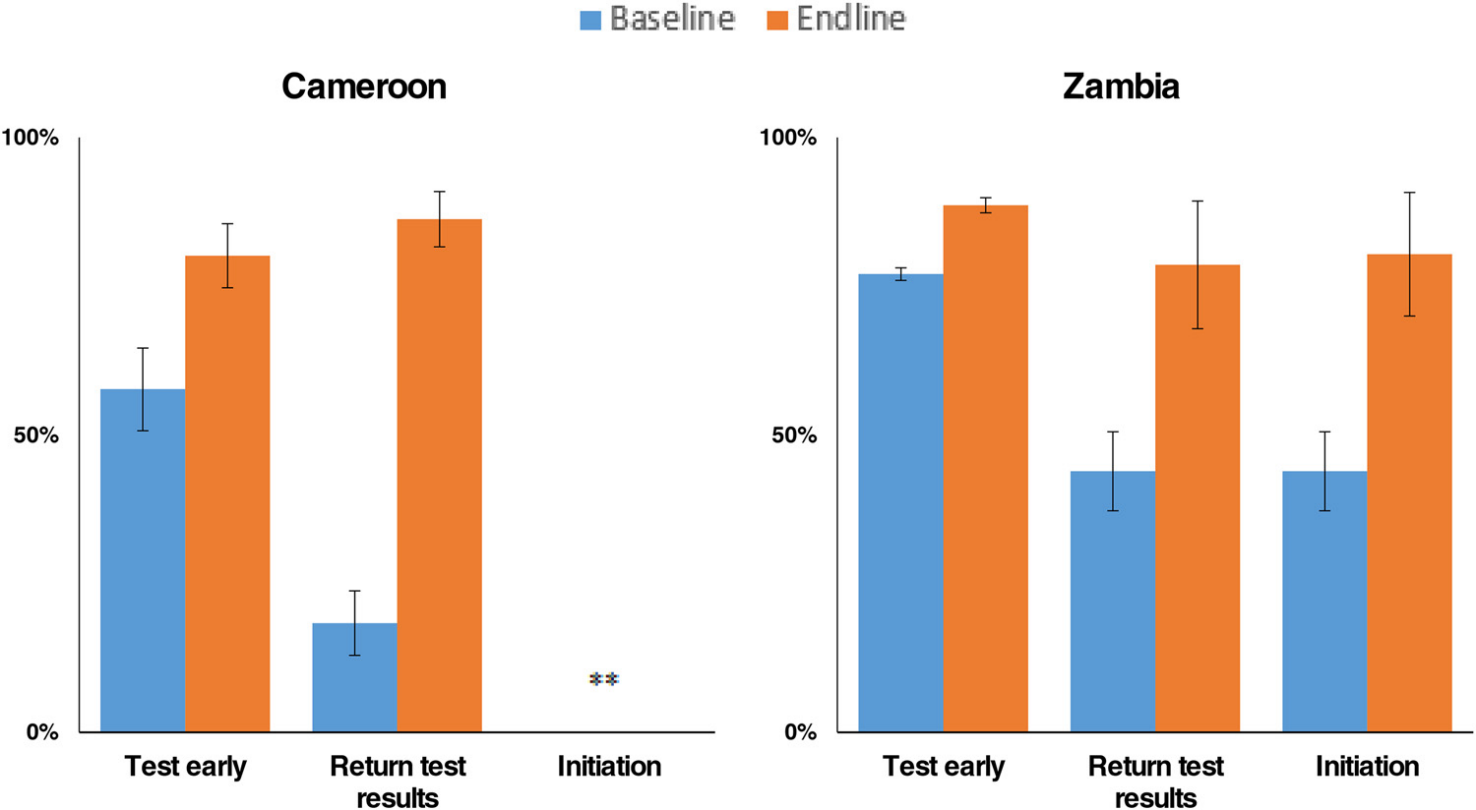
Improvements in Early Testing, Timely Return of Test Results, and Antiretroviral Initiation From Baseline to Endline After Implementing a Quality Improvement Collaborative Approach, Cameroon and Zambia

The QIC approach itself and the facility-level changes led to an improvement in performance in all 3 steps in the EID cascade: (1) early HIV testing for infants under 8 weeks of age, (2) timely return of EID results to caregivers, and (3) rapid ART initiation for infants found to be HIV infected (Tables 4 and 5, Figure 3).

**TABLE 4.** Improvements in Early Infant Diagnosis, Timely Return of Test Results, and Antiretroviral Initiation From Baseline to Endline After Implementing a Quality Improvement Collaborative Approach, Cameroon

	Indicators	Baseline Oct. 2015–Feb. 2016	Implementation Mar. 2016–Jun. 2017	Endline Feb. 2017–Jun. 2017	Change Base to End
EID testing	Eligible HEIs who received EID DBS at <8 weeks of age, %	57	71	82	24
	Eligible HEIs who received EID DBS at >8 weeks of age, %	42	29	20	−22
Return of test results	HEIs tested who were identified as HIV infected, %	2	6	4	2
	All HEIs tested whose EID DBS PCR test results were documented and shared with caregiver, %	18	81	86	68
	Mean time from facility receiving results from laboratory to results being reported to caregiver, days	19	8	3	−16
ART initiation	HIV-infected infants newly initiated on ART, %	33	51	89	56

Abbreviations: ART, antiretroviral therapy; DBS, dried blood sample; EID, early infant diagnosis; HEI, HIV-exposed infant; PCR, polymerase chain reaction.

**TABLE 5.** Improvements in Early Infant Diagnosis, Timely Return of Test Results, and Antiretroviral Initiation From Baseline to Endline After Implementing a Quality Improvement Collaborative Approach, Zambia

	Indicators	Baseline Jan. 2016–Dec. 2016	Implementation Mar. 2017–May 2018	Endline Mar. 2018–May 2018	Change Base to End
EID testing	HEIs who received EID DBS at <8 weeks of age, %	77	84	89	12
	HEIs who received EID DBS at >8 weeks of age, %				
Return of test results	HEIs tested who were identified as infected, %	3	3	2	−1
	HEIs identified as infected who received their results, %	44	86	79	35
	Mean time between positive EID DBS result and initiating ART, days	48	14	9	−39
ART initiation	HIV-infected infants initiated on ART, %	44	83	80	36
	HIV-infected infants initiated on ART the same day positive test were received, %	12	18	11	−1
	HIV-positive infants initiated on ART within 2 weeks of receiving positive test results, %	23	61	71	48

Abbreviations: ART, antiretroviral therapy; DBS, dried blood sample; EID, early infant diagnosis; HEI, HIV-exposed infant; PCR, polymerase chain reaction.

Improvement occurred in early HIV testing for infants under 8 weeks, timely return of EID results to caregivers, and rapid ART initiation for HIV-infected infants.

In Cameroon, EID testing for HEIs under 8 weeks of age improved from an aggregate performance of 57% (113 tested of 197 eligible for testing) during the 5-month baseline period to 80% (165 tested of 207 eligible for testing) during the 5-month endline period (*P*<.01). In Zambia, EID testing improved from an aggregate performance of 77% (4,773 infants under 8 weeks of age tested of 6,197 total infants tested) during the 12-month baseline period to 89% (2,144 infants under 8 weeks tested of 2,420 total infants tested) during the 3-month endline period (*P*<.01). In a comparison of the same baseline and endline periods, the return of positive test results to caregivers improved from 18% (36 test results returned of 196 total tests done) to 86% (182 test results returned of 211 total tests done) in Cameroon (*P*<.01). Return of all test results to caregivers improved from 44% (94 positive test results returned of 214 total positive tests) to 79% (44 positive test results returned of 56 total positive tests) in Zambia (*P*<.01). In Zambia, ART initiation improved from 44% (94 infants initiated on ART of 214 infants with positive HIV test results) to 80% (45 infants initiated on ART of 56 infants with positive HIV test results) (*P*<.01) and ART initiation within 2 weeks of diagnosis improved from 23% (50 infants initiated on ART of 214 infants with positive HIV test results) to 71% (40 infants initiated on ART of 56 infants with positive HIV test results) (*P*<.01). In Cameroon, the number of infants with positive HIV test results and infants initiated on ART was 1 infant at baseline to 8 infants at endline; given the small numbers, we used absolute numbers not proportions to analyze performance. Due to the very small sample size, we did not conduct statistical tests of significance because they would not have produced meaningful results.

## DISCUSSION

Despite the availability of relevant policies, guidelines, and training initiatives aimed at improving the HEI cascade in Cameroon and Zambia, quality challenges had persisted for years. We found that the use of QIC methodology enabled providers to bridge the know-do gap and was associated with substantial improvements in HEI testing coverage, return of results to caretakers, and swift linkage of HIV-infected infants to treatment in both countries.

The use of clear targets, defined processes, collaborative problem-solving, and ongoing performance evaluation were critical enablers of the successful QI collaboratives. The social dynamics of the QIC approach enhanced the sense of shared purpose and community among HCWs, fostered teamwork and friendly competition, and built leadership support while creating an internal enabling environment at the facility level, characteristics of QI projects that have been noted elsewhere.^16^^–^^18^ Quarterly learning sessions, monthly data collection, and QI mentoring encouraged the rapid and sustained improvements and facilitated diffusion of innovation. These fundamental activities provided site-level teams with consistent and supervised opportunities to identify and address ongoing challenges to program implementation while continuously measuring progress toward the aim.

The use of clear targets, defined processes, collaborative problem-solving, and ongoing performance evaluation were critical enablers of the successful QI collaboratives.

Strengths of the project included MOH leadership; the number of HFs; the magnitude and consistency of improvements in these critically important and challenging service delivery domains; the similarity of the findings in 2 countries with different HIV epidemics and EID responses; and the development of resources, methods, and tools that can be used at additional HFs.

As highlighted by Kruk et al.^37^ and the *Lancet* Global Health Commission on High Quality Health Systems, more global deaths are due to poor-quality care than insufficient access to health services, and high-quality health systems could prevent more than 8 million deaths a year in low- and middle-income countries. Investing in quality management—including the development of quality standards, measurement of quality indicators, and implementation of QI methodologies—is increasingly a priority of both MOHs and global health donors. For example, PEPFAR highlights the importance of QI methods in its guidance, funds QICs in multiple partner countries, and supported a multi-year QI capacity-building course for health ministry partners across sub-Saharan Africa.^30^ In 2018, the World Health Organization, the World Bank, and the OECD collaborated on a call to action for quality health services, calling for the development of national health care quality policies and strategies inclusive of improvement methods and interventions.^38^ Multiple MOHs have incorporated QI methods—including QICs—into national policies and guidelines, ensuring that these activities take place irrespective of donor involvement. In Mozambique, HF teams implement QI projects with support from MOH, and quarterly provincial supervision occurs routinely with or without donor support (Isabel Pereira, MD, CDC Mozambique, personal communication, 2019). In Tanzania, MOH HIV program leaders routinely perform QI-focused supportive supervision and mentoring activities independent of donor involvement.^39^

Experience shows that quality management, including the use of QI methods and tools, is a high-value and sustainable approach to health systems strengthening. Not all quality challenges require QICs, however, and identifying when a QIC is the optimal intervention is a priority for MOHs and other implementers. As above, important criteria include a high-priority quality challenge shared by multiple sites; a refractory quality challenge that has not improved following simpler interventions, such as training, quality assurance, and/or single-site QI activities; and an enabling environment including available HF staff and strong leadership support.

### Limitations

As with most QIC projects and time-series analyses, inferring causality between the intervention and the results in the current study is limited by the absence of a control or comparison group, and generalizability is limited by the nonrandom selection of HFs.^40^ However, expert consensus suggests that randomized studies of QI project effectiveness are likely to be an inappropriate study method and that statistical process control methods such as the use of run charts are the preferred approach to determining project success.^41^^,^^42^ Additional limitations of our analysis include the fact that the number of HEIs identified each month was generally quite small, making the use of percentages less informative than it would be with larger samples. It is also possible that some of the changes identified during the intervention were the result of improved data quality rather than improvements in service delivery.

QICs are a relatively resource-intensive intervention, requiring substantial time and effort on the part of HF teams and their district-level mentors. As noted, each project described in this article included stakeholder engagement, training, quarterly in-person learning sessions, and hundreds of supportive supervision visits to HFs over the 12-month project lifespan. This methodology is clearly not appropriate for all quality challenges, but in the context of a high-priority quality shortfall with a substantial know-do gap where other interventions have not succeeded, it is a critically important addition to the health systems toolkit. In the case of EID, for example, the relative cost of a time-limited QIC pales in comparison to the cost of HIV testing, prevention, and treatment services, as well as the cost of low-quality care.

## CONCLUSIONS

Despite robust evidence, supportive policies, national guidelines, and widespread training initiatives, the provision of effective testing and treatment services to HEIs has lagged in countries around the world, with dire consequences for the infants of HIV-infected women. The use of QIC methodology can effectively bridge this know-do gap by empowering HCWs to design solutions tailored to their specific settings. The well-established approach used in these projects is resource-intensive, and additional exploration may be warranted to determine if less intensive approaches can be as effective.

## Translation

**En Français**

**Améliorer les Services de Prise en Charge pour les Enfants Exposés au VIH en Zambie et au Cameroun à l'aide d'une Approche Collaborative d'Amélioration de la Qualité**

**Résultats Clés**

- Bien que les conceptions des CAQ varient pour s'adapter aux contextes locaux au Cameroun et en Zambie, elles ont utilisé des indicateurs et des méthodes similaires, et chaque CAQ a abouti à une amélioration de la couverture des tests et à un temps de retour des résultats prompte des tests pour les enfants exposés au VIH.
- L'initiation du traitement antirétroviral le même jour pour les enfants infectés par le VIH s'est améliorée en Zambie, mais le nombre des enfants infectés par le VIH au Cameroun était trop petit pour être analysé.
- La dynamique sociale de l'approche CAQ a amélioré le sens du but commun/partagés de la communauté, a favorisé le travail d'équipe et la compétition amicale, et a renforcé le soutien du leadership tout en créant un environnement interne favorable au niveau de la formation sanitaire. Les séances d'apprentissage trimestrielles, la collecte de données mensuelle et le mentorat continu pour l'amélioration de la qualité ont conduit à des améliorations importantes qui ont été maintenues pendant toute la durée de l'intervention.

**Implications clés**

- L'utilisation de la méthodologie de CAQ a permis aux agents de santé de concevoir des solutions adaptées à leurs contextes spécifiques, et chaque CAQ a abouti à un « paquet de changement » d'initiatives réussies qui ont été disséminer dans chaque pays.
- Les décideurs des politiques du secteur de la santé devraient envisager d'institutionnaliser l'approche de CAQ et de favoriser sa mise en œuvre ciblée pour relever les défis de qualité réfractaire, y compris l'inclusion des méthodes CAQ dans les politiques nationales, les directives, la formation et les systèmes de suivi.

**RESUME**

**Introduction:** Le diagnostic précoce des enfants exposes au VIH (EEVIH) et l'initiation rapide d'un traitement antirétroviral (TARV) sont des interventions vitales pour les nourrissons/enfants infectés par le VIH. Au Cameroun et en Zambie, la couverture de dépistage précoce pour les EEVIH est sous-optimale et le délai de mise en route du TARV pour les enfants infectés par le VIH dépasse souvent les normes nationales malgré de nombreuses initiatives de politiques et formations.

**Méthodes:** ICAP de l'Université de Columbia a soutenu les Ministères de la Santé du Cameroun et de la Zambie (MINSANTE) et des partenaires locaux pour mettre en œuvre des collaboratives d'amélioration de la qualité (CAQ) pour améliorer la couverture du diagnostique précoce des EEVIH et l'initiation du TARV dans 17 formations sanitaires (FOSA) au Cameroun (Mars 2016 à Juin 2017) et 15 FOSA en Zambie (Mars 2017 à Juin 2018). Dans chaque pays, le ministère de la Santé a dirigé la conception du projet et la sélection du site. Le MINSANTE et ICAP ont fourni une formation en amélioration de la qualité et des supervisions mensuelle de coaching, ce qui a permis aux équipes des FOSA de mener des analyses des causes profondes, de concevoir et de mettre en œuvre des interventions adaptées au contexte, de mener des tests rapides de changement, d'analyser les progrès mensuels et de se réunir lors de sessions d'apprentissage trimestrielles pour comparer les performances et partager les meilleures pratiques.

**Résultats:** Au Cameroun, la couverture des tests de diagnostique précoce est passée de 57% (113/197 EEVIH testés) au cours d'une période de 5 mois d'analyse situationnelle et mise en œuvre du projet à 80% (165/207) au cours de la période finale de 5 mois interventions des tests rapides de changement. En Zambie, la couverture de diagnostique précoce a amélioré 77% (4,773/6,197) au cours d'une période de 12 mois d'analyse situationnelle et mise en œuvre du projet à 89% (2,144/2,420) au cours de la période finale de 3 mois interventions des tests rapides de changement. Dans la comparaison des mêmes périodes de mise en œuvre et finales, le retour des résultats de tests positifs aux garde malades/mères est passé de 18% (36/196 garde malades/mères notifiés) à 86% (182/211) au Cameroun et de 44% (94/214) à 79% en Zambie. L'initiation du TARV est passée de 44% (94/214 nourrissons infectés par le VIH) à 80% (45/56) en Zambie; le nombre des enfants infectés par le VIH au Cameroun était trop peu pour détecter des différences significatives.

**Conclusions:** Les CAQ ont amélioré la promptitude dans l'initiation du diagnostique précoce et du TARV dans les deux pays. En plus de renforcer les capacités d'amélioration de la qualité et d'améliorer les résultats, les CAQ ont abouti à un « ensemble de changements » d'initiatives réussies qui ont été disséminer dans chaque pays.

**Peer Reviewed**

**First published online**: June 7, 2021.
